# Influencing factors and prediction methods of radiotherapy and chemotherapy in patients with lung cancer based on logistic regression analysis

**DOI:** 10.1038/s41598-022-25592-6

**Published:** 2022-12-06

**Authors:** Yuxia Liu, Chang Xu, Chengyan Xing, Mingwei Chen

**Affiliations:** 1 The Department of Respiratory and Critical Care Medicine, The First Affiliated Hospital of Xi’an Jiaotong University, 277 West Yanta Road, Xi’an, 710061 Shanxi China; 2 Library of Binzhou Medical University, Yantai, Shandong China; 3 Department of Radiology, Binzhou Medical University Hospital, Binzhou, Shandong China

**Keywords:** Cancer, Diseases, Cancer

## Abstract

Logistic regression analysis has widespread applications in clinical disease diagnosis, but it has not yet been applied to assess the acceptance of radiotherapy and chemotherapy in patients with lung cancer. A prediction model was established to investigate the influencing factors of radiotherapy and chemotherapy in lung cancer patients in order to provide useful information for clinicians to develop targeted and effective treatment. A sample was admitted of lung cancer patients to Binzhou Medical University Hospital stays from January 2020 to June 2021. After investigating doctors, nurses, patients, managers and conducting expert demonstration, the questionnaire was formed. The questionnaire was filled out by the patient or the patient's family members. The factors in the questionnaire data of patients accepting and not accepting radiotherapy and chemotherapy were compared for univariate analysis, and the significantly different single factor were analyzed by multifactor logistic regression analysis, explored the influencing factors of radiotherapy and chemotherapy in lung cancer patients established a predictive model and drew the receiver operating characteristic curve (ROC curve). The factors of two groups had statistically significant differences or no statistically significant differences. After multifactor logistic regression analysis was conducted, own personality, self-care ability, disease course classification, own attitude towards disease treatment, and family attitude towards disease treatment were included in the influencing factors of radiotherapy and chemotherapy in patients with lung cancer. Then, a predictive model was established. The area under the ROC curve of the predicted model was 0.973, the 95% confidence interval was 0.952–0.995, the optimal critical value was 0.832, the sensitivity was 91.84%, the specificity was 89.09%, and the accuracy was 90.85%. Based on logistic regression analysis, the prediction model could predict the extent of accepting radiotherapy and chemotherapy in patients with lung cancer. Understanding the factors related to patients with lung cancer accepting radiotherapy and chemotherapy could provide useful information for the targeted and effective treatment by clinicians.

## Introduction

Cancer incidence and mortality rates have been rising in China, and starting from 2010, cancer has become the number one cause of death in China and a major public health problem in the country^1^. In this past year alone, lung cancer occurred in approximately 135,000 patients, and it was responsible for 132,000 deaths in the United States^2^. Lung cancer has remained the leading cause of cancer deaths worldwide^3^.

Thus far, the four main treatments for lung cancer are surgery, radiotherapy, chemotherapy, and bio-targeted therapy^4–8^. Patients with early lung cancer, that is stages 1 and 2, could first consider surgical treatment in accordance with the treatment norms^9,10^. Adjuvant treatment after surgery and inoperable lung cancer need radiotherapy and chemotherapy^11^. According to statistics, approximately 60% of all patients with lung cancer may need to accept radiotherapy and chemotherapy, which play a very important role in the treatment of lung cancer^12^. Radiotherapy and chemotherapy could kill cancer cells and reduce the chance of spreading^13,14^. A certain course of radiotherapy and chemotherapy treatment could improve the control of the disease, and it has a positive treatment significance for delaying the development speed of the disease and improving the quality of life^15^. For various reasons such as anxiety, worry about the quality of life, many patients with lung cancer are not willing to accept radiotherapy and chemotherapy treatment^16,17^. In order to improve the quality of life of lung cancer patients, it is necessary for clinicians to make the best diagnosis and treatment plan for lung cancer patients, and to further understand the influencing factors of lung cancer patients receiving radiotherapy and chemotherapy. In some past studies, it has been shown that the most important factors affecting patients receiving chemotherapy are age, sex, financial status, whether reimbursement is provided, rural and urban of patients. Logistic regression analysis generally starts with a univariate analysis, which can provide information about the characteristics of the data distribution. The main purpose of univariate analysis is to explore the form of independent variables into the model to better describe the relationship between dependent variables and the second, some potentially meaningless variables can be eliminated to reduce the number of variables in multivariate analysis and ensure the stability and simplicity of the results. Multivariate analysis is more complex than univariate analysis, and it is favored by researchers, considering the correlation among various variables. In the present study, to understand the degree of acceptance of radiotherapy and chemotherapy for patients with lung cancer, the intention of recent patients with lung cancer to accept radiotherapy and chemotherapy was investigated. Logistic regression analysis of the influencing factors was also conducted to provide useful information for clinicians to carry out targeted and effective treatment for the benefit of more patients.

## Methods

### Setting, participants, and study design

A total of 160 patients with lung cancer who were hospitalized at the Binzhou Medical University Hospital from January 2020 to June 2021 were randomly selected. The inclusion criteria were as follows: pathologically diagnosed with lung cancer, with informed consent, and under voluntary cooperation. Patients with cognitive dysfunction were excluded. This study was approved by the Ethics Committee.

The research team consulted the literature; investigated doctors, nurses, patients, and managers; and conducted expert demonstration to form a questionnaire. The research content included whether to accept radiotherapy and chemotherapy, gender, age, cultural level, own personality, own income, acceptance of payment ratio, family economy, self-care ability, disease course classification, understanding of radiotherapy and chemotherapy, own attitude towards disease treatment, family attitude towards disease treatment, and knowledge of lung cancer. The investigation study was divided into two groups on the basis of whether to accept radiotherapy and chemotherapy. The questionnaire belongs to a descriptive study.

### Data analysis

SPSS 25.0 software was used in all statistical analyses. The observation data were counting data, represented by examples. The factors in the questionnaire data of patients accepting and not accepting radiotherapy and chemotherapy were compared for univariate analysis. χ2 test was used to compare the differences between groups. Multifactor logistic regression analysis was conducted to analyze two statistically significant groups, the influencing factors of radiotherapy and chemotherapy among patients with lung cancer were explored, and a predictive model was established. The receiver operating characteristic (ROC) curve was used to determine the predictive value of the regression model. *P* < 0.05 indicated statistically significant difference.

### Ethics approval and consent to participate

The study was reviewed and approved by the Review Boards of the Binzhou Medical University Hospital. The research methods and questionnaires used in this study were performed in accordance with the relevant guidelines of Binzhou Medical University Hospital on scientific research and the regulations of Binzhou Medical University Hospital on questionnaire survey and return visit. All participants agreed to and signed the written informed consent form prior to enrollment into the study.

## Results

### General information

In this survey, 160 questionnaires were issued, and 160 questionnaires were recovered, with a recovery rate of 100%. Upon excluding incomplete questionnaires, 153 questionnaires were analyzed, with an efficiency of 95.63%. A total of 96 questionnaires belonged to the accept group (62.75%), and 57 questionnaires belonged to the not-accept group (37.25%).

### Univariate analysis of the two groups

In the two groups of data, age, own personality, own income, family economy, self-care ability, disease course classification, understanding of radiotherapy and chemotherapy, own attitude towards disease treatment, family attitude towards disease treatment, and knowledge of lung cancer showed statistical significance (*P* < 0.05). On the contrary, gender, cultural level, acceptance of payment ratio exhibited no statistically significant difference (*P* > 0.05), as shown in Table 1.

**Table 1:** Univariate analysis of two groups data.

Factor	Type	Accept group	Not accept group	χ^2^	*p*
Gender	Male	51	27	0.474	0.491
	Female	45	30		
Age	55 ≤ Age	22	9	8.895	0.031
	55 < Age ≤ 65	39	15		
	65 < Age ≤ 75	25	18		
	75 < Age	10	15		
Culture level	Junior high school education or below	41	29	1.148	0.563
	Higher school education	38	18		
	University degree or above	17	10		
Own personality	Extravert	61	21	10.251	0.001
	Introvert	35	36		
Own income	RMB 10,000 or above	13	11	20.243	0.000
	RMB 2,000 ~ 10,000	69	21		
	RMB 2,000 or less	14	25		
Acceptance of the payment ratio	At all of your own expense	11	6	0.040	0.980
	Less below 70%	34	20		
	Less below 50%	51	31		
Family economy	Good	49	9	23.310	0.000
	Fair	34	25		
	Poor	13	23		
Self-care ability	Completely	64	15	23.359	0.000
	Partially	24	25		
	Invalid	8	17		
Disease course classification	≤ 6 months	57	4	43.381	0.000
	6 ~ 12 months	24	24		
	≥ 12 months	15	29		
Understanding of radiotherapy and chemotherapy	Fear	23	33	17.750	0.000
	No matter	73	24		
Own attitude towards disease treatment	Positive	62	10	40.350	0.000
	General	24	18		
	Negative	10	29		
Family attitude towards disease treatment	Positive	58	5	50.368	0.000
	General	24	15		
	Negative	14	37		
Knowledge of lung cancer	Early detection can cure	66	15	26.207	0.000
	Radiotherapy and chemotherapy can delay life	6	9		
	Radiotherapy and chemotherapy does not delay life	12	19		
	Radiotherapy and chemotherapy accelerated deterioration	12	14		

### Multifactor logistic regression analysis

This study established a multi-factor logistic regression model to determine whether or not to receive radiotherapy and chemotherapy is the stress variable, assigned as 1 = yes and 2 = No. The indicators of *P* < 0.05 in the univariate analysis (Table 1) were taken as independent variables. Age, own personality, and other 10 factors were taken as independent variables (variable assignment in Table 2). The regression process adopted gradual regression method and included independent variable selection and elimination. *P* > 0.05 values were excluded, while *P* < 0.05 values were selected.

**Table 2:** Multi factor assignment table.

Factor	Assignment
Gender	Male = 1; Female = 2
Age	55 ≤ Age = 1; 55 < Age ≤ 65 = 2; 65 < Age ≤ 75 = 3; 75 < Age = 4
Culture level	Junior high school education or below = 1; Higher school education = 2; University degree or above = 3
Own personality	Extravert = 1; Introvert = 2
Own income	RMB 10,000 or above = 1; RMB 2,000 ~ 10,000 = 2; RMB 2,000 or less = 3
Acceptance of the payment ratio	At all of your own expense = 1; Less below 70% = 2; Less below 50% = 3
Family economy	Good = 1; Fair = 2; Poor = 3
Self-care ability	Completely = 1; Partially = 2; Invalid = 3
Disease course classification	≤ 6 months = 1; 6 ~ 12 months = 2; ≥ 12 months = 3
Understanding of radiotherapy and chemotherapy	Fear = 1; No matter = 2
Own attitude towards disease treatment	Positive = 1; General = 2; Negative = 3
Family attitude towards disease treatment	Positive = 1; General = 2; Negative = 3
Knowledge of lung cancer	Early detection can cure = 1; Radiotherapy and chemotherapy can delay life = 2; Radiotherapy and chemotherapy does not delay life = 3; Radiotherapy and chemotherapy accelerated deterioration = 4

Regression results. The multifactor logistic regression analysis found that own personality, self-care ability, disease course classification, own attitude towards disease treatment, and family attitude towards disease treatment were factors affecting the radiotherapy and chemotherapy of patients with lung cancer. As shown in Table 3, the prediction model expression is as follows: Logit (*P*) =  − 18.785 + 1.854X1 + 1.93X2 + 1.691X3 + 2.03X4 + 2.418X5, where covariates X1, X2, X3, X4, and X5 refer to own personality, self-care ability, disease course classification, own attitude towards disease treatment, and family attitude towards disease treatment, respectively.

**Table 3:** Analysis results of logistic regression by gradual regression method.

Factor	B	Standard Error	Wald value	Degree of freedom	*P*	Exp(B)	EXP(B) 95%CI
Own personality	1.854	0.743	6.222	1	0.013	6.385	1.488 ~ 27.405
Self-care ability	1.93	0.571	11.425	1	0.001	6.886	2.249 ~ 21.082
Disease course classification	1.691	0.499	11.507	1	0.001	5.427	2.042 ~ 14.422
Own attitude towards disease treatment	2.03	0.507	16.018	1	0.000	7.617	2.818 ~ 20.586
Family attitude towards disease treatment	2.418	0.509	22.568	1	0.000	11.226	4.139 ~ 30.446
Constants	-18.785	3.376	30.961	1	0.000	0.000	-

### Comparison of ROC curves of prediction model and various factors

**Figure 1:**
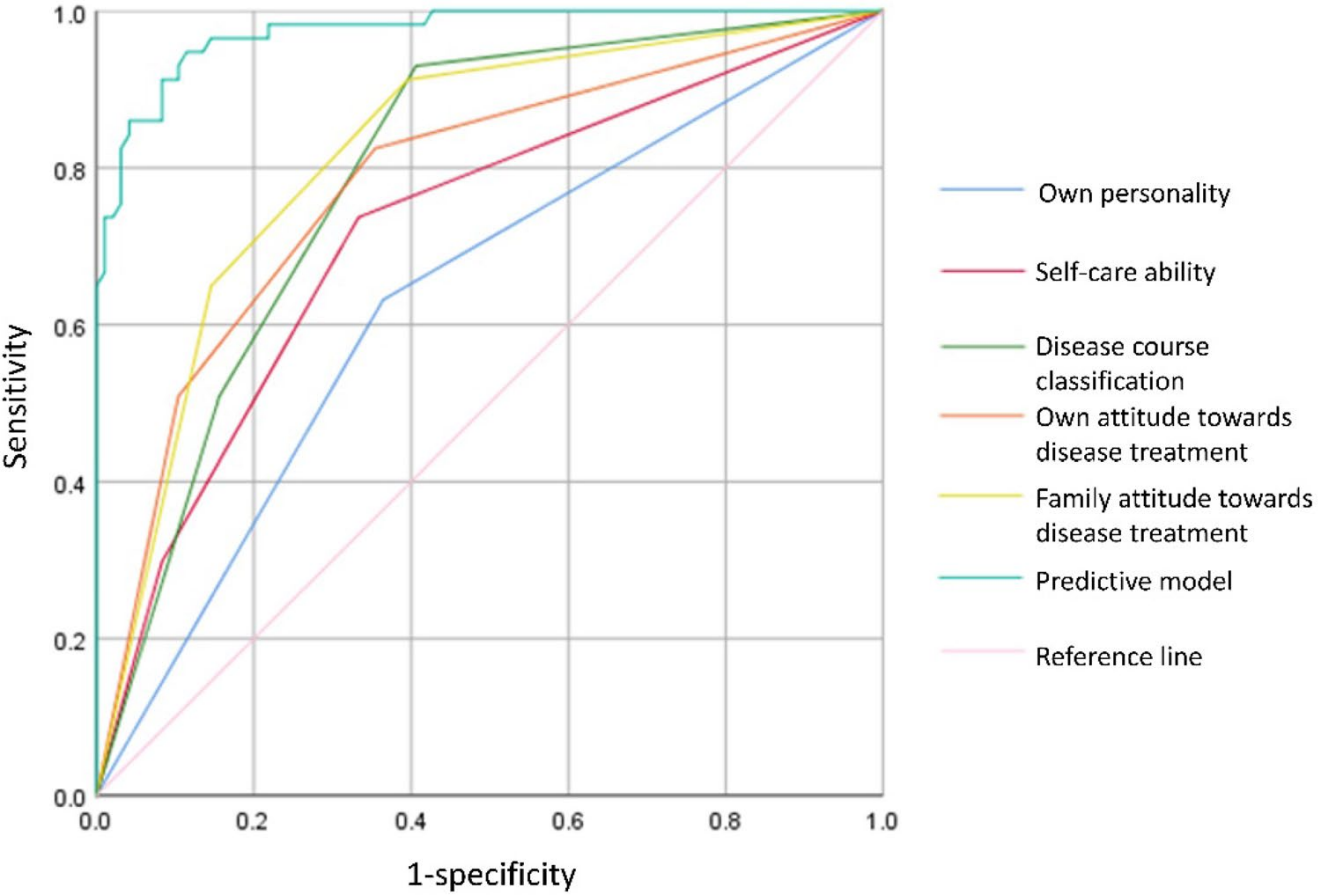
ROC curve of the predictive model and the various factors. The area under the ROC curve of the predicted model was 0.973.

The area under the ROC curve of the predicted model was 0.973, the 95% confidence interval (CI) was 0.952–0.995, the optimal critical value was 0.832, the sensitivity was 91.84%, the specificity was 89.09%, and the accuracy was 90.85%. Further details are shown in Table 4 and Fig. 1.

**Table 4:** Value of predictive model and various factors.

Factor	Optimal critical value	Sensitivity (%)	Specificity (%)	Accuracy (%)	AUC	95%CI	*P*-Value
Predictive model	0.832	91.84	89.09	90.85	0.973	0.952 ~ 0.995	0.000
Own personality	0.267	74.39	50.70	63.40	0.633	0.542 ~ 0.725	0.006
Self-care ability	0.404	68.75	68.00	68.63	0.721	0.636 ~ 0.806	0.000
Disease course classification	0.524	74.31	65.91	71.90	0.792	0.721 ~ 0.864	0.000
Own attitude towards disease treatment	0.471	75.44	74.36	75.16	0.782	0.705 ~ 0.86	0.000
Family attitude towards disease treatment	0.516	80.40	72.55	77.78	0.82	0.751 ~ 0.889	0.000

## Discussion

As a clinically important treatment means and strategy, the radiotherapy and chemotherapy for lung cancer, similar to other treatment methods, has advantages and disadvantages^18,19^. They reflect the basic principles of multidisciplinary comprehensive treatment of tumors^20^. Patients have the greatest clinical benefit, which could not only quickly remove peripheral circulating cancer cells and hidden metastatic metastases but also rapidly reduce local primary lesions to relieve local oppression and local invasion symptoms and improve the quality of life of patients^21^. Clinically, the side effects of radiotherapy and chemotherapy could not be underestimated, including the so-called body injury side, such as eating difficulties caused by radioactive esophagitis^22^, cough and breathing difficulties caused by radioactive pneumonia^23^, abnormal brain cognitive behavior caused by radiotherapy and chemotherapy^24^, and gastrointestinal dysfunction^25^. Doctors need to weigh the pros and cons clinically, combined with the situation of the family members, and make calm and rational choices rather than the same synchronization^26^. A specific individualized treatment plan must be established in accordance with each patient’s own situation, psychological condition, the disease itself, and the specific situation of the family members..

Univariate analysis of the two groups of data showed statistical significance in age, own personality, own income, family economy, self-care ability, disease course classification, understanding of radiotherapy and chemotherapy, own attitude towards disease treatment, family attitude towards disease treatment, and knowledge of lung cancer (*P* < 0.05), whereas no statistical difference was found in gender, cultural level, and acceptance of payment ratio (*P* > 0.05). The findings demonstrated that these three factors have no effect on whether or not patients with lung cancer accept radiotherapy and chemotherapy. Further logistic regression analysis of factors with statistical significance found that five indicators, such as own personality, self-care ability, disease course classification, own attitude towards disease treatment, and family attitude towards disease treatment, were retained in the regression model (*P* < 0.05). Age, income, family economy, understanding of radiotherapy and chemotherapy, and knowledge of lung cancer were excluded. In the past 10 years, the level of population education in China has made a new and relatively significant leap, and we can gain more "population quality dividend" in the era of the popularization of higher education. The improvement of people's education level has enhanced the understanding of disease treatment, and given full play to their own initiative.

In Chinese custom, a previous preference for men over women exists. However, in this study, gender was not an influencing factor, indicating that Chinese people today have basically eliminated the preference for men over women and treat both genders equally. Age was also not an influencing factor, indicating that the idea of respecting and loving the elderly in traditional social culture has always existed and that treatment has not been given up because of their old age, different from the African diaspora^27^. All of the above factors were not affected by the level of knowledge and culture. Therefore, cultural level was excluded. Acceptance of payment ratio, own income, and family economy were three factors related to treatment cost, which was not an influencing factor as it did not affect whether or not patients with lung cancer accept radiotherapy and chemotherapy. Treatment cost is related to the improvement of people’s living standards, the substantial growth of gross national product, and the implementation of national medical care since the reform and opening up. The understanding of radiotherapy and chemotherapy and the knowledge of lung cancer were not included, indicating that treating a disease is necessary, seeing a doctor is deeply rooted in the hearts of sick people, and the people’s awareness of seeking medical treatment is gradually improved^28^. Logistic analysis showed that among the five factors, own personality, which is a psychological factor; self-care ability; disease classification, which is the disease itself; and own and family attitudes towards disease treatment further affected the decision of patients with lung cancer to accept radiotherapy and chemotherapy factors. The above factors could help clinicians determine further treatment.

The limitations of the study are based on a small sample size belonging to a population of cultural background (China). If the same study were conducted in the populations of other countries of different cultural, economic, and social environments, then the results would be different outcomes. It would be impactful if such studies and models were used for similar objectives in different parts of the world to have a more wholesome understanding of influencing factors. In the future, we will further expand the sample size, increase the depth and breadth of the questionnaires, introduce more effects into the study, and improve the reliability of the conclusions.

## Conclusions

The five indicators (including own personality, self-treatment ability, disease course classification, own attitude towards the treatment of disease, and family attitude towards the treatment of disease) are the main factors for accepting radiotherapy and chemotherapy treatment among patients with lung cancer. The predictive model based on logistic regression analysis could better predict the extent of accepting radiotherapy and chemotherapy. Understanding the factors related to patients with lung cancer could provide useful information for the targeted and effective treatment by clinicians.

## Supplementary Information

Supplementary Information.

## Data Availability

Datasets are available in the manuscript. Any additional information and data are available upon reasonable request. The data and materials can be shared with corresponding author upon reasonable request.

## Abbreviation

ROC: Receiver operating characteristic
CI: Confidence interval
