# Supplementary material for: Influencing factors and prediction methods of radiotherapy and chemotherapy in patients with lung cancer based on logistic regression analysis
Source: Sci Rep. 2022 Dec 6;12:21094. doi: 10.1038/s41598-022-25592-6 (PMC9726881; doi:10.1038/s41598-022-25592-6)
Supplement: Supplementary file 1 — Supplementary Information. [file 41598_2022_25592_MOESM1_ESM.docx]

Questionnaire on chemoradiotherapy acceptance and influencing factors in lung cancer patients

Whether you receive chemoradiotherapy：（ ）1. Accept；2：Not-accept

Gender：（ ）1. Male；2. Female

Age：（ ）1. 55≤Age；2. 55<Age≤65；3. 65<Age≤75；4. 75<Age

Culture level：（ ）1. Junior high school education or below；2. Higher school education；3. University degree or above

Own personality：（ ）1. Extravert；2. Introvert

Own income：（ ）1. RMB 10,000 or above；2. RMB 2,000~10,000；3. RMB 2,000 or less

Acceptance of the payment ratio：（ ）1. At all of your own expense；2. Less below 70%；3. Less below 50%

Family economy：（ ）1. Good；2. Fair；3. Poor

Self-care ability：（ ）1. Completely；2. Partially；3. Invalid

Disease course classification：（ ）1. ≤6 months；2. 6~12 months；3. ≥12 months

Understanding of radiotherapy and chemotherapy：（ ）1. Fear；2. No matter

Own attitude towards disease treatment：（ ）1. Positive；2. General；3. Negative

Family attitude towards disease treatment：（ ）1. Positive；2. General；3. Negative

Knowledge of lung cancer：（ ）1. Early detection can cure；2. Radiotherapy and chemotherapy can delay life；3. Radiotherapy and chemotherapy does not delay life；4. Radiotherapy and chemotherapy accelerated deterioration
